# Program-wide review and follow-up of erythema Induratum of Bazin and tuberculosis-associated ocular inflammation management in a TB low-incidence setting: need for improved treatment candidate selection, therapy standardization, and care collaboration

**DOI:** 10.1186/s12879-019-3737-5

**Published:** 2019-01-29

**Authors:** William J. Connors, Dina A. Fisher, Dennis Y. Kunimoto, Julie M. Jarand

**Affiliations:** 1 Department of Medicine, University of Calgary, Alberta, Canada; 2 Calgary Tuberculosis Services, Alberta, Canada; 3 Edmonton Tuberculosis Program, Alberta, Canada; 4 Faculty of Medicine and Dentistry, University of Alberta, Alberta, Edmonton Canada; 5 Foothills Medical Centre, Rm 303, 3rd Floor North Tower, 1403, 29th Street, NW, Calgary, Alberta T2N 2T9 Canada

**Keywords:** Tuberculosis, Erythema induratum, Tuberculosis-associated ocular inflammation, Treatment outcome, Canada, Tuberculids, Uveitis

## Abstract

**Background:**

Erythema induratum of Bazin (EIB) – nodular vasculitis associated with *Mycobacterium tuberculosis* (TB) – and Tuberculosis-Associated Ocular Inflammation (TB-AOI) represent uncommon manifestations of TB. There is limited data and a lack of diagnostic and treatment standards for these conditions.

**Methods:**

Eleven-year retrospective review of EIB and TB-AOI cases managed in a provincial TB program with prospective phone-based follow-up of anti-tubercular therapy (ATT) recipients. Presumptive TB-AOI and EIB diagnoses were determined by ophthalmologist or dermatologist assessments correlated with positive tuberculin skin test and/or QuantiFERON-TB Gold, along with pathologic criteria in EIB cases.

**Results:**

Of 21 EIB and 20 TB-AOI cases that received ATT, 13 and 11, respectively, were reached for follow-up. The majority of EIB and TB-AOI cases were female and immigrated from TB high-burden countries. Median durations of pre-diagnosis symptoms were 2 and 0.8 years (IQR 2.5 & 1.1) for EIB and TB-AOI cases, respectively. Overall, 14 different ATT regimens were used for a median duration of 6 months (range 5–9). ATT related adverse events resulting in treatment discontinuation occurred in 14% of EIB and 10% of TB-AOI cases. On last follow-up, 76% of EIB and 42% of TB-AOI had improvement or resolution of disease.

**Conclusion:**

EIB and TB-AOI were uncommon presentations receiving variable therapy. While treatment response was modest for EIB cases, TB-AOI cases had sub-optimal treatment outcomes. The unique diagnostic and management challenges presented by these conditions in TB low-incidence settings highlight a need for improved treatment candidate selection, therapy standardization, and cross-specialty medical collaboration.

**Electronic supplementary material:**

The online version of this article (10.1186/s12879-019-3737-5) contains supplementary material, which is available to authorized users.

## Background

Tuberculosis (TB) represents a major global health problem with estimates of 2–3 billion people latently infected with tuberculosis and 10 million incident cases of tuberculosis disease globally in 2017 [1]. Although TB primarily causes pulmonary disease, 18–30% of cases in the United States and Canada involve either isolated or concurrent extra-pulmonary disease [2, 3]. Cutaneous and ocular forms of TB disease represent only a very small proportion of cases [4, 5]. In addition to direct infection of an extra-pulmonary site, a subset of tuberculosis-associated disorders may also be characterized by immune-mediated inflammatory changes, often in the absence locally detectable *Mycobacterium tuberculosis* complex. When believed to be related to a hypersensitivity reaction to TB antigens such conditions have been referred to as tuberculids, and more recently tuberculosis-associated ocular inflammation (TB-AOI) when involving the eye [4, 6, 7].

Erythema induratum of Bazin (EIB) is a tuberculid form of lobular panniculitis often occurring on the arms or legs with a clinical appearance similar to erythema nodosum but distinguished by characteristic nodular vasculitis on pathology [8, 9]. TB-AOI often shares clinical features with uveitis but may involve almost any eye structure and typically is characterized by granulomatous inflammation on pathology [10, 11]. While EIB and TB-AOI represent distinct clinical entities, they pose similar diagnostic and treatment challenges to a TB program, often resulting in empiric treatment of presumed cases, and for this reason were combined in our study.

The posited immune-mediated and/or and pauci-bacillary nature of EIB and TB-AOI makes their diagnosis reliant on non-specific clinical findings, indirect TB testing with tuberculin skin test (TST) and/or interferon gamma release assay, and, when tissue can be obtained, non-specific pathologic findings. As mycobacterial culture and molecular testing are frequently non-diagnostic or not available, a therapeutic trial of anti-tubercular therapy (ATT) for presumed cases is often pursued [7, 12]. Currently there are no defined standards for management of EIB or TB-AOI and proposed diagnostic criteria for these conditions [7, 13] have neither achieved consensus across medical specialties and few have been evaluated in TB low-incidence settings [14]. Evidence guiding ATT for these conditions predominantly come from observational studies in TB high burden countries often with limited or no clinical follow-up [6, 15–17]. The extended duration of standard TB treatment, associated healthcare costs, and potential for ATT associated adverse events further complicate treatment decisions for these conditions. The cost-benefit calculus of treating these conditions is even less favorable in TB low-incidence settings where the relative prevalence of diseases that mimic these conditions and rates of false-positive TB diagnostics may be higher.

Canada is a TB low-incidence country with a national case incidence rate of 4.7 per 100,000 population in 2013. Alberta, the fourth most populous province in Canada, has an above national average TB case incidence rate of 5.1 [18]. In this setting our study aimed to evaluate referral trends, clinical management patterns and long-term outcomes for EIB and TB-AOI cases managed in a provincial TB program. The goal of the study was to inform the development of standardized management protocols for these conditions and to address a gap in knowledge about the epidemiology and management of these conditions in TB low-incidence settings.

## Methods

### Study population

Alberta’s Tuberculosis Program operates under a public health mandate providing provincial TB screening, prevention, and exclusive community treatment services via multiple community clinics to a population of approximately 4.2 million. A program-wide database search, using diagnosis codes, identified EIB and TB-AOI cases between January 1, 2004 and December 31, 2014.

### Case definitions

TB-AOI cases were defined as having an inflammatory eye disease based on ophthalmologist assessment and a positive TST and/or QuantiFERON-TB Gold In-Tube Test® (QFT) result. EIB cases were defined as having lobular panniculitis based on dermatologist assessment, compatible pathologic findings, if biopsy had been done, and a positive TST and/or QFT result. Both EIB and TB-AOI cases were defined as being in the absence of an alternative diagnosis or concurrent active TB disease at another site. Non-tubercular causes of uveitis and erythema nodosum were screened for as indicated by clinical assessment. TST results were interpreted according to Canadian Tuberculosis Standards criteria [3] and positive QFT was defined according to manufacturer guidelines [19].

### Chart review data collection

Cases meeting definition had data collected on demographics (gender, age, country of origin), TB history (prior exposure, diagnosis, treatment), BCG history, TB diagnostics (QFT result, TST induration, chest x-ray), duration of symptoms, and treatment details (ATT type and duration, severe adverse drug events length of follow-up, clinical outcome). Severe adverse drug events were defined as those requiring ATT discontinuation. Primary and emergency phone contact information was collected to facilitate follow-up.

### Phone-based follow-up

A single researcher (WJC) conducted all phone surveys using a standardized script of questions with categorical answers relating to current EIB/TB-AOI symptom status, course of illness post treatment, and response and tolerance to prior ATT (Additional file 1). Phone contact was attempted on three different weekdays during both day and evening hours prior to listing a case as unable to be contacted. Professional phone-based translation services were used for surveys with non-English speakers.

### Analysis

Chi-squared or Fisher exact tests and two-sample *t*-test were used for comparison of categorical and continuous variables, respectively. Significance was defined as an alpha ≤0.05 in two-tailed distribution. Analysis was performed using StatPlus:mac Pro version 5 (AnalystSoft Inc., USA).

## Results

### Demographics and case characteristics

A total of 22 EIB and 20 TB-AOI cases were identified over the 11-year study period. Demographic and case characteristics are summarized in Table 1. A majority of cases in both groups had emigrated from TB high burden countries (74% overall) with the Philippines, Vietnam, and India representing the most common countries of origin overall, together accounting for 44% of cases. History of BCG vaccination was documented in 6 cases of each EIB and TB-AOI (27 and 29%, respectively).

**Table 1:** Demographics and case characteristics of EIB and TB-AOI cases - Alberta, 2004–14

	EIB (*n* = 22)	TB-AOI (*n* = 20)
Female gender – no. (%)	17 (77)	11 (55)
Age – years		
Median (IQR)	43 (8.3)	38 (17.8)
Country of origin – no. (%)		
TB-HBC*	18 (82)	14 (70)
History of latent TB^†^− no. (%)	4 (18)	4 (20)
Duration of Symptoms – months (years)	*n* = 19	*n* = 15
Median	24 (2)	9 (0.8)
IQR	30 (2.5)	13 (1.1)
Diagnostics^‡^		
TST	*n* = 16	*n* = 16
Range - induration (mm)	10–30	12–40
Median (IQR)	18 (7)	17.5 (7.8)
IGRA	*n* = 13 (7 had TST)	*n* = 15 (11 had TST)
Positive result – no. (%)	12 (92)	13 (87)
Chest X-ray	*n* = 22	*n* = 20
TB changes^§^ – no. (%)	4 (18)	1 (5)
Tissue Biopsy Pathology^#^	*n* = 17	
Granulomatous inflammation	15 (88)	No samples obtained for
Necrobiosis	9 (53)	pathology
Panniculitis	10 (59)	
Vasculitis	8 (47)	

*TB-HBC = TB High Burden Country as per WHO classification [40]

^†^TB history = reported prior exposure, documented TST conversion (positive after a prior negative), or prior LTBI therapy

^‡^TST = tuberculin skin test, IGRA = interferon-gamma release assay (QuantiFERON-TB Gold In-Tube Test)

^§^TB changes (no.) = upper lobe calcification (2), multilobar ‘infiltrate’ (1), nodule (1), hilar lymphadenopathy (1)

Pathology sums to > 100% as multiple patterns present in some specimens, No AFB or organisms seen in any samples

Seven EIB and 11 TB-AOI cases had both TST and QFT performed and discordant results (negative QFT) were reported in 1 (14%) and 2 (18%) cases, respectively. None of the discordant case had documentation of BCG vaccination. Among EIB cases, the majority had bilateral lower extremity involvement (86%) and underwent diagnostic tissue punch biopsy (77%). Detailed ophthalmologic exams and TB-AOI eye localization was not consistently documented in the TB program medical charts. Mycobacterial stains and cultures were negative for all EIB tissue biopsies. Granulomatous inflammation was the most common pathologic pattern in EIB tissue samples (88%) with all samples demonstrating granulomatous and/or panniculitis changes. Among TB-AOI cases, a single tissue sample was obtained (vitreal aspirate) which was smear and culture negative.

### Treatments and clinical outcomes

Table 2 summarizes treatment characteristics and clinical outcomes. Severe adverse drug events related to ATT - resulting in treatment discontinuation - occurred in 3 (14%) of EIB cases and 2 (10%) of TB-AOI cases. Among those completing therapy, 14 different ATT regimens were used (10 for EIB, 4 for TB-AOI). Six-months of isoniazid/rifampin with the first 2-months including pyrazinamide was most commonly used for EIB cases (32%), while six-months isoniazid/rifampin was most commonly used for TB-AOI cases (71%) (Additional file 2 & Additional file 3). Notably only one TB-AOI case received ethambutol as part of their regimen, a local practice pattern of avoiding ethambutol thought to reflect concerns about potential increased ocular toxicity in TB-AOI. Thirty-eight ATT regimens (92%) were documented as intended for active TB disease therapy, the remainders were intended for LTBI. Of 20 EIB cases with documented end of therapy clinical outcomes, a majority (*n* = 16, 80%) had resolution or improvement of clinical signs. One EIB case was lost to follow-up after initiating therapy. End of therapy clinical outcomes were only documented for a minority of TB-AOI cases (*n* = 6, 30%) of which half demonstrated clinical improvement and the remainder had no change.

**Table 2:** Treatment and clinical outcomes of EIB and TB-AOI cases - Alberta, 2004–14

	EIB (*n* = 21)	TB-AOI (*n* = 20)
Type of treatment		
Directly observed	13	5
Self-administered	8	15
Duration ATT – month		
Median (range)	6 (1–9)	6 (1–9)
Severe adverse drug events^a^ (ADE)– no. (%)	3 (14)	2 (10)
Last clinical assessment outcome – no. (%)	*n* = 20	*n* = 6
Resolved	14 (70)	0
Improved	2 (10)	3 (50)
No-change	4 (20)	3 (50)

^a^Major ADE = treatment discontinued (no.) - hepatotoxicity (1), rash (1), GI upset (2), severe fatigue (1)

### Phone-based follow-up

Thirteen EIB (62% of total) and 11 TB-AOI (55% of total) cases were reached for follow-up. Phone-based survey responses are summarized in Table 3. Of the 17 cases (8 EIB, 9 TB-AOI) not reached for follow-up: 12 no longer resided at listed contact, translation services were not available for three, and two declined participation. Compared to non-participants, the demographic and clinical characteristics of phone-based survey participants were similar (*p* > 0.05).

**Table 3:** Phone-based survey of treatment outcomes of EIB and TB-AOI cases - Alberta, 2004–14

	EIB (*n* = 21)	TB-AOI (*n* = 20)
Phone Follow-up – no. (%)	*n* = 13 (62)	*n* = 11 (55)
Time post ATT – month (year)		
Median	61 (5.1)	49 (4.1)
IQR	34 (2.8)	48 (4)
Responses		
“Do you still have *(EIB/TB-AOI)*?”		
Resolved – no. (%)	8 (62)	2 (18)
Better	2 (15)	3 (28)
Worse	0 (0)	0 (0)
No change	3 (23)	6 (54)
“How did your condition respond to the ATT?”		
Min (< 25% improvement)	1 (8)	8 (73)
Mod (~ 50%)	1 (8)	1 (9)
Significantly (~ 75%)	2 (15)	0 (0)
Complete (100%)	7 (54)	2 (18)
“How did you tolerate the ATT?”		
Poorly	5 (38)	3 (28)
Well	5 (38)	2 (18)
No issue	3 (24)	6 (54)

EI – erythema induratum, TB-AOI – tuberculosis-associated ocular inflammation, ATT – anti-tubercular therapy

### Overall disease outcomes and treatment comparisons

Overall 33 participants (21 EIB, 12 TB-AOI) who received ATT had either clinical or phone-based follow-up outcomes recorded. Of these, based on status at last contact (either clinical or phone-based), 16 EIB (76%) and five TB-AOI (42%) cases had improvement or resolution of their condition while the remainder reported no change or worsening. Demographics and clinical characteristics of those who had condition improvement or resolution compared to those with those that had no change or worsening were not significantly different for both EIB and TB-AOI groups. Similarly, no significant differences in treatment outcomes were found.

per degree of base-line TST induration (≥15 mm versus <15 mm) for either EIB or TB-AOI cases. Comparing outcomes of those who completed ATT treatment regimens containing three or more anti-TB drugs for a total of six or more months (including regimens consisting of pyrazinamide for 2 months along with two additional drugs for ≥6 months) with those that did not, outcomes were not significantly different for either EIB or TB-AOI cases (*p* > 0.05) [analysis excluded those individuals who discontinued ATT secondary to adverse drug events]. Further specific treatment effectiveness statistical comparisons were limited by the high variability in ATT regimens used and small sample size.

## Discussion

Erythema induratum of Bazin and TB-AOI represented uncommon referrals to our provincial TB program during the period of study. Most cases were among immigrants from TB high-burden countries, and the treatments provided were variable. In addition to novel epidemiological and long-term outcome data on these conditions in a TB low-incidence setting, our study highlights key programmatic challenges in the diagnosis and management of these conditions. While 76% of EIB cases had improvement or resolution of symptoms at last contact, similar responses were reported by less than half of TB-AOI cases. These findings underscore a need for improved diagnostics and clinical decision tools to enhance treatment candidate selection, particularly for suspected TB-AOI cases. Furthermore, the variable treatments used for these conditions in our program significantly limits effectiveness evaluation and illustrates a need for treatment standardization if practice improvement is to be achieved moving forward. Small sample size and treatment heterogeneity in this case series means that no conclusions about treatment effectiveness can be made.

Compared with previously published case series, the demographic characteristics of EIB and TB-AOI cases in our series are similar; females, 30–50 years of age, born or living in TB endemic settings (> 20 cases per 100,000 population per year) predominate across case series [6, 12, 14, 15, 17, 20–22]. In prior series, ATT associated clinical improvement of EIB skin lesions has ranged from 69 to 86% [20, 21], while visual acuity improvement and/or ocular inflammation reduction in TB-AOI has ranged from 24 to 85% [6, 12, 15, 17, 22]. Treatment outcomes may be influenced by delays in diagnosis, particularly in TB-AOI where prompt initiation of ATT may improve visual prognosis [22]. Although our treatment outcomes were not dissimilar from previous case series, the large range, particularly for TB-AOI, and substantial residual non-response rates, illustrate the need for improvements in the management of these conditions.

### Improved treatment candidate selection

The non-specific nature of EIB and TB-AOI clinical syndromes and a frequent lack of confirmatory diagnostics make treatment candidate selection for these conditions particularly challenging. Whereas tissue sampling for histopathology and microbiology evaluation may aid diagnostic differentiation in cases of suspected EIB [16], the low diagnostic yield and added risk of morbidity with ocular tissue sampling limits its utility for TB-AOI [11]. The integration of molecular diagnostics, criteria based histopathologic/ophthalmologic assessments, and novel imaging techniques have the potential to increase diagnostic specificity for these conditions.

Tuberculosis molecular diagnostics (e.g. nested polymerase chain reaction (PCR)) appear to have an emerging role in EIB and TB-AOI diagnosis. The reported sensitivity of ocular fluid PCR for TB has varied considerably across cases series in TB high-incidence settings, ranging from 31 to 47% in a pair of studies in India and Singapore [23, 24] to as high as 77% in a more recent Indian series using a novel multi-targeted PCR [25]. False positive rates of 5 to 9% have been reported in ocular fluid sample PCR from disease control groups [23, 26]. Tissue-based PCR results in EIB have demonstrated equally variable sensitivity with TB isolation ranging from 14 to 77% across cases series [7]. A single case-control series in China found that 32% of panniculitis and 5% of control biopsies demonstrated TB on PCR [27]. Over the period of our study, PCR for TB was not routinely done on tissue biopsies specimens that did not show evidence of acid-fast bacilli on pathology, as is common in EIB and was the case in all of our study samples. Continued limited availability, lack of standardization, and low sensitivity of these tests remain as obstacles to their wide spread use, particularly in low-incidence settings were validation studies are lacking [7, 28].

In the absence of confirmatory molecular diagnostics, histopathology in EIB may significantly aid treatment candidate selection. Lobular panniculitis with vasculitis - some authors consider veins and venules to be predominantly involved [8, 9] – and progression to tissue necrosis and granuloma formation with disease evolution are characteristic findings in EIB [7]. That relatively few EIB biopsies in our series demonstrated vasculitis, 48% compared with 90% in a large series by Segura et al. [9], may in part reflect biopsy technique and timing. In our series, punch biopsies, which often contain less subcutaneous fat [29], were most commonly obtained. Excisional biopsies with adequate subcutaneous fat - the site most commonly demonstrating vasculitis changes [9] - may optimize diagnostic yield in EIB [7]. Further, biopsies in our series may have been predominantly from later stage lesions – participants reported lesions being present a median of 2 years prior to diagnosis - which may contain less extensive vasculitis changes compared with earlier stage lesions [7]. To maximize diagnostic yield, excisional biopsies of early stage skin lesions should be preferentially utilized in cases of suspected EIB.

In the case of TB-AOI, low culture yield of ocular aspirates and potential risks associated with ocular tissue biopsy place diagnostic emphasis on ophthalmological examinations. Findings of choroidal granulomas, occlusive retinal vasculitis, and multifocal serpiginoid choroiditis have been proposed as specific to TB-AOI in high TB incidence settings [13]; however, the transferability of these findings to low incidence setting has been questioned [28]. Serial ophthalmologic examinations also may serve a diagnostic function following initiation of ATT therapy. The authors of two larger case series reported that most TB-AOI cases demonstrated a reduction of ocular inflammation within 2–6 weeks of starting ATT and quiescence by six months [15, 30]. More recently, circumventing the low yield and risks of ocular tissue biopsy, several researchers have proposed a role for positron emission tomography/CT (^18^F-FDG-PET/CT) to identify extra-ocular sites of infection for tissue sampling [31]. Evaluating a series of 20 TB-AOI cases, Doycheva et al. identified FDG-uptake in mediastinal and hilar lymph nodes in 45% of cases and 40% of those who underwent biopsy cultured TB [32]. The Collaborative Ocular Tuberculosis Study (COTS)-1 group is working to address the lack of evidence for the diagnostic approaches to TB-AOI and highlight the importance of multipronged approach in a recent study evaluating treatment outcomes [33].

### Therapy standardization

While variations in case definitions (e.g. inclusion of cases with concurrent active TB disease, differing or absent pathologic criteria used for EIB cases) and outcome measurements (e.g. timing of post treatment follow-up, use of serial ophthalmologic exams for TB-AOI) limit cross-series ATT comparisons. Acknowledging these limitations, a trend of positive clinical response and relapse reduction is apparent with extended duration triple-drug ATT for both EIB and TB-AOI.

Cases-series evaluating EIB treatments have shown higher non-response and relapse rates among cases receiving mono- or dual- drug ATT compared with those receiving triple-drug ATT for at least 6 months [20, 21]. Similar increased effectiveness with triple-drug ATT has been shown for TB-AOI with several studies supporting extension of treatment to 9 or more months [17, 22]. A case-control study by Ang et al. in Singapore demonstrated improved one-year post treatment visual acuity and relapse reduction among TB-AOI cases that received greater than 9 months of ATT [15]. Similarly, a retrospective review of TB-AOI by Agrawal et al. in the United Kingdom found TB-AOI patients who received more than 9 months of triple-ATT had a lower likelihood of treatment failure [12]. While a significant difference in overall disease outcomes between ATT regimens was not found in our case series, small sample size and a high rate of loss to follow-up, particularly among TB-AOI cases, restricted our evaluation. In keeping with findings of prior EIB case series [16, 20], the two cases with post treatment relapse in our series had not received triple-drug ATT regimens (Additional file 2).

Whether EIB and TB-AOI represent immune-mediated response to LTBI or are manifestations of subclinical TB disease continues to be an area of scientific debate with implications for the selection of appropriate treatment [7, 10]. Despite this uncertainty, informed by the observational study results outlined above, a number of authors favor treating these conditions similar to active TB disease using standard drug-susceptible TB ATT regimens recommended by the Centers for Disease Control and Prevention [34]: isoniazid, rifampicin, ethambutol, and pyrazinamide for 2 months followed by isoniazid, rifampicin for at least 4 additional months for EIB [5, 7, 16] with potential extension to 7 plus months for TB-AOI [12]. Supporting such active TB disease therapy for EIB, a Japanese review of 66 cases over a 20 year period found that that a quarter of EIB cases also had active TB lymph node disease [35]. In the case of TB-AOI, the high morbidity associated with delayed or incomplete treatment of potential ocular TB disease as well as concern about limited drug penetration to the eye, favor prolonged active TB disease ATT therapy [15, 36]. Despite this, treatment remains highly variable, particularly in low incidence settings, with only 58% of suspected TB-AOI cases receiving standard drug-susceptible TB ATT in a recent US series [6].

### Limitations

The medical records used for the retrospective component of this study did not contain detailed ophthalmologic exam findings from baseline and follow-up. While the majority of TB-AOI cases were designated as ‘uveitis’ (90%), further information on location, extent and nature of ocular inflammation could not be reliably captured. Previous studies have shown variable ATT response between predominantly anterior, posterior or panuveitis cases of TB-AOI [6, 12, 15]; analysis of treatment response by such subgrouping was not possible in our study. Furthermore, lack of serial ophthalmological exam findings to objectively quantify ATT response limits our TB-AOI clinical outcome measures. Our prospective outcome measures for both EIB and TB-AOI were based on unstandardized subjective patient report limiting generalization and potentially introducing recall bias. Use of standardized classifications systems, such as that proposed by the International Uveitis Study Group in the case of TB-AOI [37], is needed to improve cross study comparison in this field. Finally, unreliable capture of corticosteroid prescription data in are TB program records made us unable to comment on what, if any, effect their adjunctive use may have had. It should be noted that adjunctive use of corticosteroids has not been supported in EIB and findings are conflicting for TB-AOI; case series level evidence suggesting benefit in early TB-AOI disease [30, 38] needing to be balanced against findings of increase likelihood of relapse [12].

### Cross-specialty medical collaboration

As highlighted by limitations of the present study and the outlined challenges in treatment candidate selection, collaboration between TB specialist and ophthalmologist for TB-AOI or dermato-pathologists for EIB is needed to optimize care for these conditions. This may be particularly true in low incidence setting where non-tuberculosis providers may be unfamiliar or uncomfortable managing these conditions. Supporting this need for collaboration, a recent report from the American Uveitis Survey found that ophthalmologists in TB low-incidence countries were much more likely to defer TB-AOI treatment decisions to an infectious disease or TB specialist compared with those in high TB high-incidence countries (76 versus 34%) [39]. While there are emerging collaborative models for TB-AOI management in TB low-incidence settings, such as the UK [12] and US [6], to our knowledge no such models have been reported for EIB.

## Conclusions

While erythema induratum of Bazin and TB-AOI represent uncommon manifestations of TB they pose unique management challenges, particularly in TB low-incidence settings. As highlighted by our study and review of current research in this area, improvements in treatment candidate selection and therapy standardization are needed to establish the optimal management of these conditions. Cross-specialty collaborative management of these conditions along with prospective multi-site research collaborations are needed to ensure these uncommon conditions are better understood and effectively managed as part of TB elimination efforts.

## Additional files

Additional file 1: Phone Interview. Standardized script used for follow-up participant phone interviews. (DOCX 15 kb)

Additional file 2: Erythema induratum of Bazin treatment and outcomes**.** Tabular summary of treatment details, clinical outcome, and phone-interview patient reported outcome. (DOCX 18 kb)

Additional file 3: Tuberculosis-Associated Ocular Inflammation treatment and outcomes. Tabular summary of treatment details, clinical outcome, and phone-interview patient reported outcome. (DOCX 18 kb)

## Abbreviations

ADE: Adverse drug event
ATT: Anti-tuberculous therapy
BCG: Bacille Calmette-Guerin
CT: Computed tomography
EIB: Erythema induratum of Bazin
FDG: Fluorodeoxyglucose
IQR: Interquartile range
LTBI: Latent tuberculosis infection
PCR: Polymerase chain reaction
PET: Positrone emission tomography
QFT: *QuantiFERON*-TB Gold
TB: Tuberculosis
TB-AOI: Tuberculosis associated ocular inflammation
TST: Tuberculin skin test
UK: United Kingdom
US: United States
