# Supplementary material for: Program-wide review and follow-up of erythema Induratum of Bazin and tuberculosis-associated ocular inflammation management in a TB low-incidence setting: need for improved treatment candidate selection, therapy standardization, and care collaboration
Source: BMC Infect Dis. 2019 Jan 29;19:97. doi: 10.1186/s12879-019-3737-5 (PMC6352374; doi:10.1186/s12879-019-3737-5)
Supplement: Supplementary file 1 — Phone Interview. Standardized script used for follow-up participant phone interviews. (DOCX 15 kb) [file 12879_2019_3737_MOESM1_ESM.docx]

**Additional file 1**

**Phone Interview Script**

“Since the conclusion of your care through the Tuberculosis Program:

1. Do you still have the skin / eye condition you were treated for? Y/N
   1. If yes, how is it now compared to before you received treatment? Better / Worse / No change
2. If your skin condition showed a positive response to the tuberculosis treatment, how much improvement did you see?

1= minimal (~25%), 2= moderate (~50%), 3= significant (75%), 4= complete (100%)

1. If you had complete resolution of skin condition following therapy, have you had subsequent recurrence? Y / N
2. Do you remember having difficulty with the medicines you received as part of your treatment?

1= poorly tolerated/unable to continue, 2= well tolerated/but some side-effect, 3 = no side-effects

1. If you had side-effect, please describe:”

______________________________________________________________________________
