# Supplementary material for: Program-wide review and follow-up of erythema Induratum of Bazin and tuberculosis-associated ocular inflammation management in a TB low-incidence setting: need for improved treatment candidate selection, therapy standardization, and care collaboration
Source: BMC Infect Dis. 2019 Jan 29;19:97. doi: 10.1186/s12879-019-3737-5 (PMC6352374; doi:10.1186/s12879-019-3737-5)
Supplement: Supplementary file 2 — Erythema induratum of Bazin treatment and outcomes. Tabular summary of treatment details, clinical outcome, and phone-interview patient reported outcome. (DOCX 18 kb) [file 12879_2019_3737_MOESM2_ESM.docx]

**Additional file 2**

**Erythema induratum of Bazin treatment and outcomes**

| Patient No. | Treatment | | | Follow-up | |
| --- | --- | --- | --- | --- | --- |
|  | Anti-tubercular therapy (ATT)*  (months) † | Total Duration  (months) | Outcome  (clinical) | Time post ATT - months (years) | Outcome  (reported) |
| 1 | (6)HRZ | 6 | RESOLVED | 19 (1.6) | RESOLVED |
| 2 | (2)HRZ(4)HR | 6 | RESOLVED | 23 (1.9) | RESOLVED |
| 3 | (1)HRZE(1)HRZ(4)HR | 6 | RESOLVED | 20 (1.7) | RESOLVED |
| 4 | (1)HRZ | 1‡ | RESOLVED | 42 (3.5) | RELAPSE |
| 5 | (2)HRZ(4)HR | 6 | RESOLVED | 46 (3.8) | RESOLVED |
| 6 | (2)HRZ(4)HR | 6 | RESOLVED | 66 (5.5) | IMPROVED |
| 7 | (2)HRZ(4)HR | 6 | IMPROVED | 65 (5.4) | RESOLVED |
| 8 | (2)HRZ(4)HR | 6 | RESOLVED | - | - |
| 9 | (2)HRZE(5)HRE | 5§ | RESOLVED | 61 (5.1) | RESOLVED |
| 10 | (1)HRZE(8)HRE | 9 | RESOLVED | 93 (7.8) | RESOLVED |
| 11 | (2)HRZE(4)HRE | 6 | RESOLVED | - | - |
| 12 | (2)HRZE(4)HRE | 6 | RESOLVED | - | - |
| 13 | (2)HRZE(4)HRE | 6 | RESOLVED | - | - |
| 14 | (7)HR | 7 | RESOLVED | 105 (8.8) | RELAPSE |
| 15 | (2)HRZE(4)HR | 6 | UNCHANGED | - | - |
| 16 | (2)HRZE(4)HRE | 6 | UNCHANGED | - | - |
| 17 | (2)HRZ(4)HR | 6 | IMPROVED | 100 (8.3) | RESOLVED |
| 18 | (2)HRZE(4)HR | 6 | UNCHANGED | - | - |
| 19 | (1)HRZE🡪(8)QE | 9 | UNCHANGED | 76 (6.3) | IMPROVED |
| 20 | (2)HR | 2¶ | - | 60 (5) | UNCHANGED |
| 21 | (6)HRZE | 6 | RESOLVED | - | - |

*ATT abbreviations: E – ethambutol, H - isoniazid, Q – quinolone (levofloxacin or moxifloxacin) R – rifampin, Z - pyrazinamide

† “(months)” is in reference to following medications which were concurrently administered unless “🡪” present, designating switch in regime

‡ Treatment discontinued because of rash

§ Treatment discontinued because of gastro-intestinal upset

¶ Treatment discontinued because of hepatotoxicity
