# Supplementary material for: Program-wide review and follow-up of erythema Induratum of Bazin and tuberculosis-associated ocular inflammation management in a TB low-incidence setting: need for improved treatment candidate selection, therapy standardization, and care collaboration
Source: BMC Infect Dis. 2019 Jan 29;19:97. doi: 10.1186/s12879-019-3737-5 (PMC6352374; doi:10.1186/s12879-019-3737-5)
Supplement: Supplementary file 3 — Tuberculosis-Associated Ocular Inflammation treatment and outcomes. Tabular summary of treatment details, clinical outcome, and phone-interview patient reported outcome. (DOCX 18 kb) [file 12879_2019_3737_MOESM3_ESM.docx]

**Additional file 3**

**Tuberculosis-Associated Ocular Inflammation treatment and outcomes**

| Patient No. | Treatment | |  | Follow-up | |
| --- | --- | --- | --- | --- | --- |
|  | Anti-tubercular therapy (ATT)*  (months)† | Total Duration  (months) | Outcome  (clinical) | Time post ATT  -months (years) | Outcome (reported) |
| 1 | (6)HR | 6 | - | - | - |
| 2 | (1)HRZ🡪(9)H | 10 | - | - | - |
| 3 | (1)HRQZ | 1‡ | IMPROVED | 87 (7.3) | RESOLVED |
| 4 | (1)HRZ | 1§ | UNCHANGED | 81 (6.8) | UNCHANGED |
| 5 | (6)HRZ | 6 | IMPROVED | 21 (1.8) | RELAPSE |
| 6 | (2)HRQZ(4)HRQ | 6 | UNCHANGED | - | - |
| 7 | (2)HRZ(4)HR | 6 | UNCHANGED | 34 (2.8) | IMPROVED |
| 8 | (1.5)HRZE🡪(4)QR | 5.5¶ | - | 57 (4.8) | UNCHANGED |
| 9 | (6)HR | 6 | - | - | - |
| 10 | (6)HR | 6 | - | 83 (6.9) | RESOLVED |
| 11 | (6)HR | 6 | - | - | - |
| 12 | (6)HR | 6 | - | - | - |
| 13 | (6)HR | 6 | - | 52 (4.3) | UNCHANGED |
| 14 | (6)HR | 6 | - | - | - |
| 15 | (6)HR | 6 | - | 49 (4.1) | UNCHANGED |
| 16 | (6)HR | 6 | - | - | - |
| 17 | (1)HR🡪(9)RQ | 10 | IMPROVED | - | - |
| 18 | (6)HR | 6 | - | 8 (0.7) | IMPROVED |
| 19 | (6)HR | 6 | - | 21 (1.8) | UNCHANGED |
| 20 | (6)HR | 6 | - | 10 (0.8) | IMPROVED |

* ATT abbreviations: E – ethambutol, H - isoniazid, Q – quinolone (levofloxacin or moxifloxacin) R – rifampin, Z - pyrazinamide

† “(months)” is in reference to following medications which were concurrently administered unless “🡪” present, designating switch in regime

‡ Treatment discontinued because of gastro-intestinal upset

§ Treatment discontinued because of abdominal pain

¶ Treatment discontinued because of severe fatigue
